# Synergism of Co/Na in BiVO_4_ microstructures for visible-light driven degradation of toxic dyes in water†

**DOI:** 10.1039/d3na00048f

**Published:** 2023-03-27

**Authors:** Muhammad Zeeshan Abid, Khezina Rafiq, Abdul Rauf, Syed Shoaib Ahmad Shah, Rongchao Jin, Ejaz Hussain

**Affiliations:** a Institute of Chemistry, Inorganic Materials Laboratory 52S, The Islamia University of Bahawalpur-63100 Pakistan ejaz.hussain@iub.edu.pk +92 3026500254; b Department of Chemistry, School of Natural Sciences, National University of Sciences and Technology Islamabad-24090 Pakistan; c Department of Chemistry, Carnegie Mellon University Pittsburgh Pennsylvania-15213 USA rongchao@andrew.cmu.edu

## Abstract

In this work, we report a synergism of Co/Na in Co@Na–BiVO_4_ microstructures to boost the photocatalytic performance of bismuth vanadate (BiVO_4_) catalysts. A co-precipitation method has been employed to synthesize blossom-like BiVO_4_ microstructures with incorporation of Co and Na metals, followed by calcination at 350 °C. The structure and morphology of the as-prepared photocatalysts are characterized by XRD, Raman, FTIR, SEM, EDX, AFM, UV-vis/DRS and PL techniques. Dye degradation activities are evaluated by UV-vis spectroscopy, in which methylene blue, Congo red and rhodamine B dyes are chosen for comparative study. The activities of bare BiVO_4_, Co–BiVO_4_, Na–BiVO_4_, and Co@Na–BiVO_4_ are compared. To evaluate the ideal conditions, various factors that affect degradation efficiencies have been investigated. The results of this study show that the Co@Na–BiVO_4_ photocatalysts exhibit higher activity than bare BiVO_4_, Co–BiVO_4_ or Na–BiVO_4_. The higher efficiencies were attributed to the synergistic role of Co and Na contents. This synergism assists in better charge separation and more electron transportation to the active sites during the photoreaction.

## Abstract 2

In this work, we report a synergism of Co/Na in Co@Na–BiVO_4_ microstructures to boost the photocatalytic performance of bismuth vanadate (BiVO_4_) catalysts.

## Graphical abstract

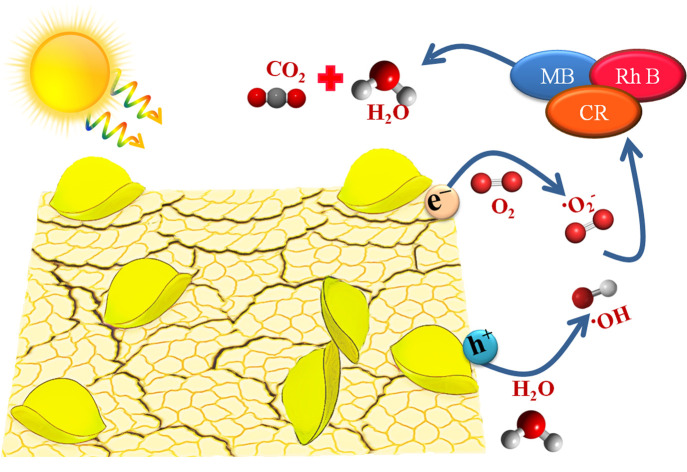

## 1. Introduction

Water is the main natural resource covering over 70% of the earth's surface. The accessibility of safe drinking water (0.5%) is crucial not only for humans but also for the survival of other living organisms. Annually, 200 000 tons of toxic dyes are dumped into water bodies.^1^ The textile, paper, leather, paint and cosmetics industries are the major contributors of these contaminants (*i.e.* toxic dyes). Such dyes are hazardous, carcinogenic and non-biodegradable effluents which cause damage to human health, plant species, environment and soil even at very low concentrations (*i.e.* less than 1 ppm).^2^

Common ways to remove the dyes from water are through adsorption and coagulation.^3^ However, these methods produce secondary pollutants because dyes are only changed from the liquid to the solid phase during adsorption and coagulation.^4^ To resolve this issue, an environment-friendly and cost-effective approach is urgently required that can utilize visible light for the removal of organic dyes from aqueous solutions.^5^ Photocatalysts accelerate the photoreactions and convert photons into chemical energy. Photocatalysts are now widely used in agriculture, medicine, electrical appliances, photoelectrochemical reactions, environmental and energy fields. A number of metal-element-doped semiconductor materials have been employed for visible light induced dye degradation for water purification purposes.^6^

BiVO_4_ and TiO_2_ are considered the most advantageous photocatalysts because of their photocatalytic activity, stability and non-toxicity. However, TiO_2_ only responds to ultraviolet radiations, which restricts its further application.^7^ In this regard, photocatalysts that can efficiently work in the visible range are more desired. Unfortunately some visible light responsive photocatalysts have stability issues; for example, CdS is active under visible light but not stable in an aqueous system due to photocorrosion.^8^ Among the photocatalysts, BiVO_4_ has been reported to be a promising candidate.^9^ Various techniques have been introduced for the development of BiVO_4_ nanostructures, including the solid-state reaction,^10^ hydrolysis of metal alkoxides,^11^ co-precipitation^12^ and hydrothermal synthesis.^13^ Nanostructures obtained by different methods often have different crystal structures and defects that influence the photocatalytic efficiencies. Among the common synthetic protocols, the co-precipitation method was found to deliver higher yields with high purity without using organic solvents.^14^ Moreover, this strategy is cost effective and straight forward.

One of the common problems with BiVO_4_ is the rapid recombination of photogenerated electrons with holes, which reduces the overall activities. However, this problem could be reduced by generating more active centers in the form of transition metal dopants. Similarly, the BiVO_4_ activity can also be enhanced by doping with non-metals, metals, metal cations and non-metal anions or by fusion with another oxide. For combination with BiVO_4_, noble metals such as Pt, Au, Pd and Ag serve as excellent cocatalysts in photocatalysis.^15^ But due to high cost, these metals are less common in dye degradation applications, although these metals enhance electron–hole separation (due to electron quenching abilities) and increase light absorption. The separation of charge carriers and their lifetime can also be enhanced by developing composites *via* incorporating suitable dopants. Many composites of metals or metals oxides, such as Au, CeO_2,_ WO_3_, CdS, SnO_2_ and TiO_2_, still have some drawbacks and their photocatalytic efficiencies are still unsatisfactory.^16–19^ Among the dopants, cobalt (Co) doped^20,21^ and alkali (Na^+^ and K^+^) doped BiVO_4_ nanostructures have been reported for better charge transfer in photocatalysis.^22,23^

To demonstrate the advantage of an alkali metal oxide (Na_2_O) with a transition metal co-catalyst, Co and Na contents were *in situ* employed and incorporated into BiVO_4_. This work was designed to promote the charge transfer from the semiconductor (BiVO_4_) to the co-catalyst active sites by shifting the Fermi level. The Co in the form of Co_3_O_4_ acts as excellent active sites for dye degradation. Another advantage of Co is to enhance the photon absorption surfaces of the semiconductor support. The approach employed in this study exhibits various other advantages like the excellent stability of the synthesized photocatalysts, higher dispersion rather than agglomeration and excellent dye degradation efficiencies. This work demonstrates the extended absorption in visible light that attributes to higher dye degradation efficiencies of various toxic dyes (*e.g.* methylene blue, Congo red and rhodamine B) under solar radiations.

## 2. Experimental

### 2.1 Materials used

The following chemicals were used for the synthesis of photocatalysts, bismuth nitrate pentahydrate (Bi(NO_3_)_3_·5H_2_O, Sigma Aldrich 98%), ammonium metavanadate (NH_4_VO_3_, Sigma Aldrich 98%), cobalt(ii) nitrate hexahydrate (Co(NO_3_)_2_·6H_2_O, Sigma Aldrich 98%), sodium hydroxide (NaOH, Sigma Aldrich 98%) and sodium borohydride (NaBH_4_, Sigma Aldrich 98%). To monitor the degradation activities, methylene blue (MB) dye (C_16_H_18_ClN_3_S, Sigma Aldrich), Congo red (CR) dye (C_32_H_22_N_6_Na_2_O_6_S_2_), and rhodamine B (C_28_H_31_ClN_2_O_3_, Sigma Aldrich) were used.

### 2.2 Catalysts preparation

BiVO_4_ was synthesized by the co-precipitation method with optimized timescale strategy. Briefly, Bi(NO_3_)_3_·5H_2_O solution (7 mM) was prepared by dissolving 3.396 g of Bi(NO_3_)_3_·5H_2_O (Sigma Aldrich, 99%) in 50 mL of 4 M HNO_3_ solution. In the next step, NH_4_VO_3_ solution (7 mM) was prepared by adding 0.819 g of NH_4_VO_3_ (Sigma Aldrich, 99%) to 50 mL of 2 M NH_4_OH solution. To remove the dissolved oxygen, both solutions were purged with high purity argon (Ar) for 30 min. After that, the Bi(NO_3_)_3_·5H_2_O solution was added dropwise into the NH_4_VO_3_ solution with vigorous stirring for 12 h while maintaining the pH = 9 at a temperature of 10 °C. The precipitate mixture was then sonicated for 20 min by adjusting the sweep frequency (37 kHz, 300 W). The yellow precipitate was filtered out using high grade filter paper (WHA 1001325, Grade-1). The solid product was washed thoroughly with deionized water and then with absolute ethanol. After that, the product was dried at 94 °C overnight in an oven (Sanyo/MOV-112 Japan).^24^ For the synthesis of Co@Na–BiVO_4_ microstructures, an optimized amount of BiVO_4_ (155 mg) powder was transferred into a three-neck, round bottom flask, followed by addition of 50 mL of high purity deionized water (PIAS-GW1-Z) to produce a uniform slurry. After 15 min of purging with argon, Co and Na ions (from Co (NO_3_)_2_·6H_2_O & NaOH/NaCl) were dispersed and *in situ* incorporated into BiVO_4_ by using a chemical method. The amounts of Co and Na metal contents were fixed at 1 : 1 and overall 2% metal contents (2% w/w). To grow the Co@Na–BiVO_4_ microstructures, the above mixture solution was under optimized stirring (150 rpm) at approximately 10 °C for 24 h. The final suspension was filtered and dried at 94 °C. The synthesis of Co–BiVO_4_ and Na–BiVO_4_ are discussed in the ESI.†

### 2.3 Catalysts characterization

UV-vis/DRS for the as synthesized photocatalysts was obtained over the wavelength range of 265–850 nm on a PerkinElmer (λ-850+/Tungsten-Halogen) spectrophotometer. Powdered XRD analysis was conducted on an advanced XRD system (Bruker D2-phaser) equipped with a LYNXEYE XE-T Detector, 220 V/60 Hz. Using the Scherer equation, particle sizes were measured having *D* ≈ 0.9*λ*/(*β* cos *θ*); the Cu Kα operational X-ray source is (*λ* = 1.54 Å, 40 kV, 40 mA). The 2*θ* range was fixed from 15° to 80° (step: 0.05° and scan rate: 2° min^−1^), Fourier transform infrared (FT-IR) analysis was performed on a BrukerTensor-27. The SEM results were obtained using an FEI-Nova NanoSEM-450 electron microscope. The elemental composition of Co@Na–BiVO_4_ NPs was obtained using a SEM equipped with an energy dispersive X-ray (EDX) accessory. The AFM results were obtained using an Agilent 5500 SPM/AFM. The photoluminescence results were recorded on a spectrometer (LS-45, PerkinElmer). Photocatalytic dye degradation efficiencies were recorded using a UV/vis-spectrophotometer (PerkinElmer/λ-365).

### 2.4 Photocatalytic degradation experiments

To investigate comparative efficiencies, degradation experiments of selected dyes (MB, CR and RhB) were performed on a spectrophotometer (PerkinElmer/λ-365). Dye degradation experiments were performed in a Pyrex reactor of size 6.1 × 4.3 × 4.2 inches (100 mL/silicon septum, ChemGlass AF-0528-06). For photocatalytic experiments, the photoreaction set up was made to elucidate the photolysis reaction of dyes, pure BiVO_4_, Na–BiVO_4_, Co–BiVO_4_ and Co@Na–BiVO_4_ microstructures. Photocatalytic activities of all as-synthesized photocatalysts were investigated using direct sunlight. All photoreactions have been carried out on consecutive days under a clear sky to ensure accurate photon absorption. The average photon flux was measured to be 1.388 × 10^3^ W m^−2^ using a light meter (Extech/LT-300 USA). For each photoreaction, 10 mg of the photocatalyst was optimized, and treated with 50 mL of 5 ppm solution of each dye. Before starting the photoreaction, the analyte solution was stirred in the absence of light for 30 min to soak the dye with photocatalysts. At regular intervals, 3.5 mL reaction mixture was pipetted out and centrifuged to get a clear solution for UV-vis measurements.

## 3. Results and discussion

**Fig. 1:**
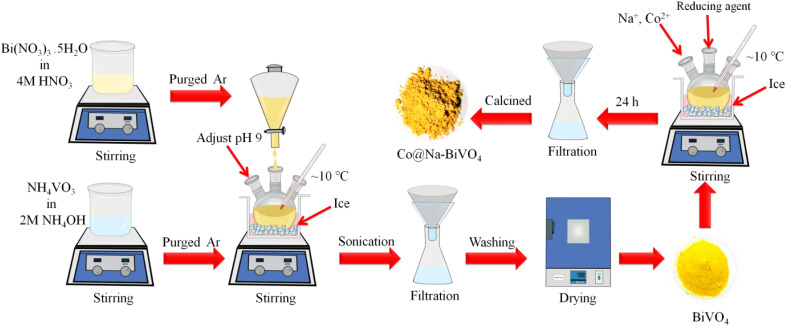
Scheme employed for the synthesis of BiVO_4_ and Co@Na–BiVO_4_.

The synthesis scheme of all photocatalysts is demonstrated in Fig. 1 (see details in the Experimental section). To remove the impurities and enhance the crystallinity, the as-synthesized photocatalysts were calcined at 350 °C for 3 h.

### 3.1 XRD

**Fig. 2:**
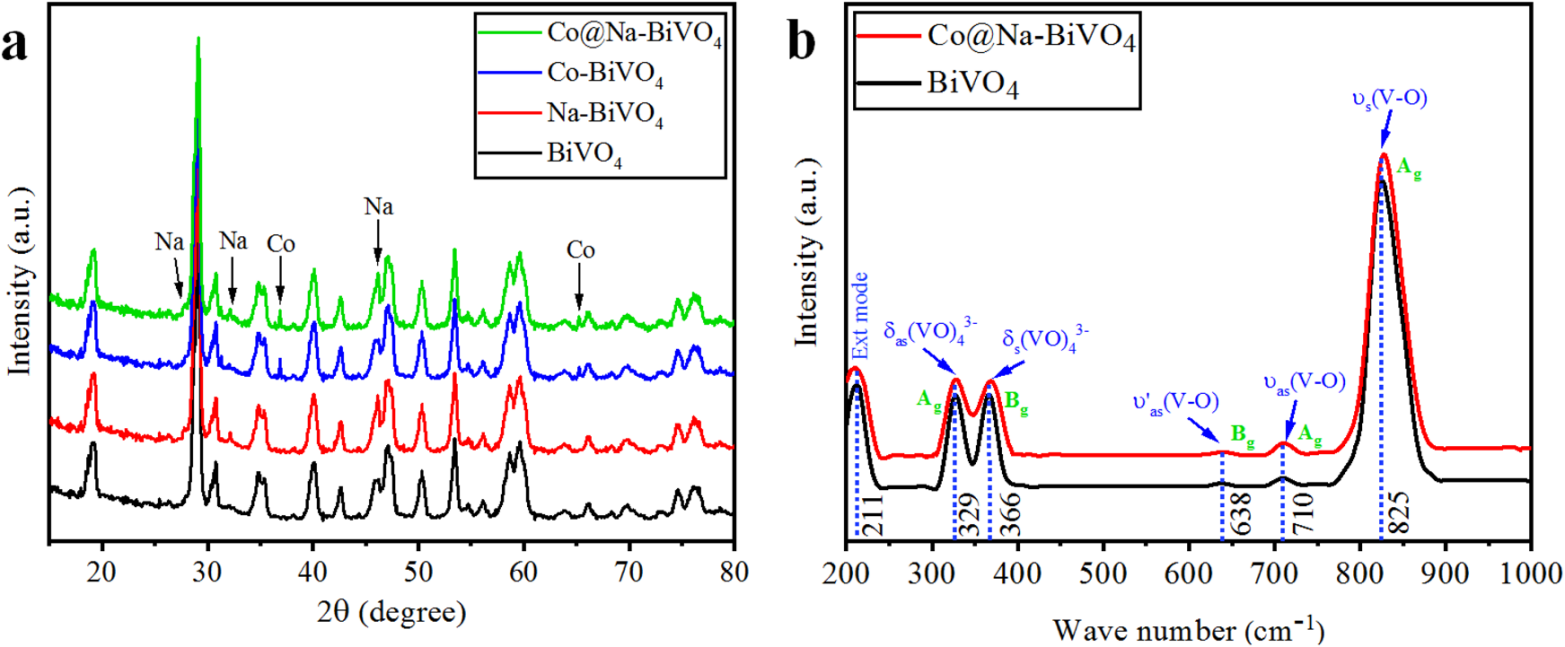
(a) Powder XRD patterns of BiVO_4_, Na–BiVO_4_, Co–BiVO_4_ and Co@Na–BiVO_4_ (b) Raman vibrations of BiVO_4_ and Co@Na–BiVO_4_ microstructures.

XRD is an excellent tool for the detection or identification of crystal phases in solid-state materials.^25^ The XRD patterns of the as-synthesized plain BiVO_4,_ Na–BiVO_4_, Co–BiVO_4_, and Co@Na–BiVO_4_ are shown in Fig. 2(a). The synthesized BiVO_4_ is monoclinic in phase (*c.f.* JCPDS#14-0688), with XRD peaks observed at 18.66°, 18.98°, 28.94°, 30.54°, 34.49°, 35.22°, 39.78°, 42.46°, 46.71°, 47.30°, 50.31°, 53.31° and 58.53°, corresponding to (110), (011), (121), (040), (200), (002), (211), (051), (240), (042), (202), (161) and (321) crystallographic planes, respectively.^25^ No other diffractions were seen, confirming the phase purity of BiVO_4_. Co metal exists in the form of cobalt oxide (Co_3_O_4_, cubic phase), with major diffraction peaks at 36.8 and 65.2° (PDF#43-1003). The ionic radius of Na^+^ (1.02 Å) is almost same as that of the Bi^3+^ ion (1.03 Å), which favors the substitution of Na^+^ on Bi^3+^, while most of the Na exists as sodium oxide Na_2_O (cubic), with major peaks observed at 27.7, 32.1 and 46.1°, in accordance with PDF#23-0528. By employing the reported method, the crystallite size was measured to be 19.7 nm. The results showed that intrinsic catalytic activity of the photocatalyst is not a sole factor of particle sizes; however, the rate of dye degradation is mainly due to metal cocatalysts over BiVO_4_ surfaces.^26^

### 3.2 Raman spectroscopy

The structure, chemical composition and bonding of the as-synthesized materials were further analyzed by Raman spectroscopy as illustrated in Fig. 2(b). The vibration bands observed at 211, 329, 366, 638, 710, and 825 cm^−1^ were attributed to the VO_4_ tetrahedron. The strong vibrations at 825 cm^−1^ were allocated to the V–O symmetric stretching mode (A_g_). At 710 and 638 cm^−1^, weak asymmetric stretching bands (A_g_ and B_g_) of V–O were observed. The asymmetric and symmetric bending vibrations of the VO_4_ tetrahedron were observed at 327 and 367 cm^−1^, respectively. In Co@Na–BiVO_4_, the intense vibration mode shifted to 828 cm^−1^ from 826 cm^−1^. In the presence of Co and Na, a shift was observed due to the decrease in the V–O bond length. Moreover, Raman band intensities and the FWHM also shortens: this behavior corresponds to crystallinity or flaws.^27,28^

### 3.3 SEM and EDX

**Fig. 3:**
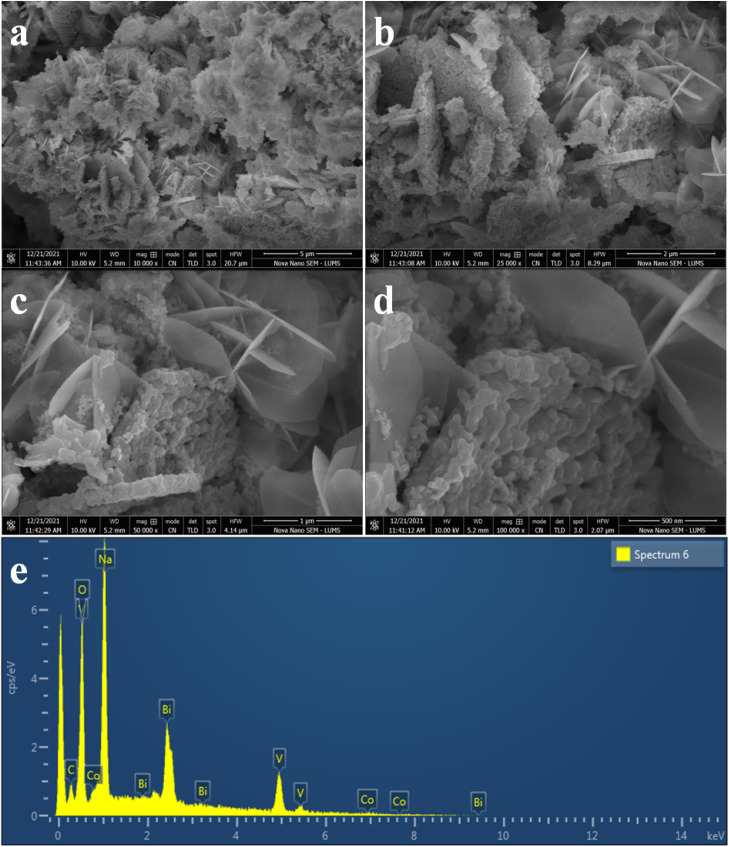
SEM images of Co@Na–BiVO_4_ microstructures (a) 5 μm (b) 2 μm (c) 1 μm and (d) 500 nm, whereas (e) represents the EDX analysis.

The structural and surface morphology of the Co@Na–BiVO_4_ photocatalysts were investigated by scanning electron microscopy (SEM). Fig. 3(a–d) shows the typical SEM images of the Co@Na–BiVO_4_ photocatalysts. The microstructures are blossom-like due to our new synthesis, rather than the conventional structures.^29^ The SEM results are in good agreement that Co (in the form of Co_3_O_4_) and Na (in the form of Na_2_O) enhance the surface area of BiVO_4_. The novelty of our work is that the blossom-like structures were developed using the time-scaled low temperature co-precipitation method (24 h, 10 °C), which led to an enhancement in the surface area and active sites. An EDX accessory was used to elucidate the elemental composition of the as-synthesized Co@Na–BiVO_4_ photocatalysts Fig. 3(e), which confirms the presence of Co and Na contents with BiVO_4_. The weights and atomic percentages of elements are tabulated in Table S1.†

### 3.4 AFM

**Fig. 4:**
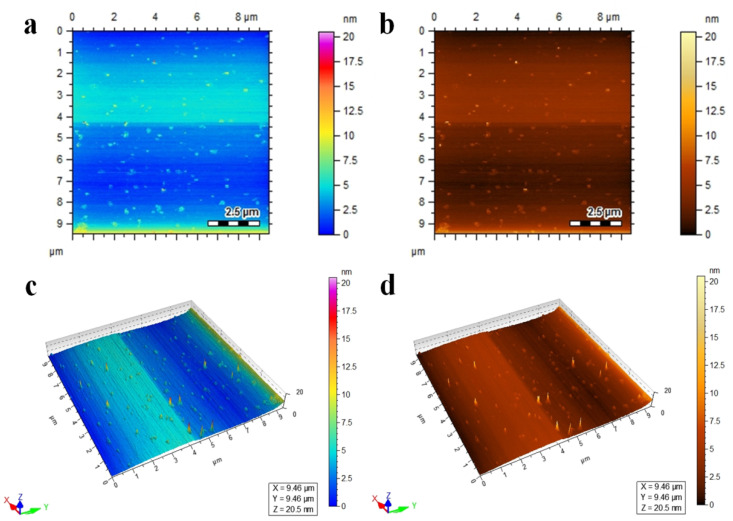
AFM images of Co@Na–BiVO_4_ (a and b) topographical images, and (c and d) three-dimensional view.

Atomic force microscopy (AFM) is a characterization technique that provides information about the topography, surface roughness, size distribution, and morphology of materials at a high resolution.^30^ The Co@Na–BiVO_4_ photocatalysts were studied using AFM; the two-dimensional and three-dimensional topographical images are shown in Fig. 4(a–d). The scanning area is 9 × 9 μm, The AFM results revealed the surface morphology of Co@Na–BiVO_4_ particles with an average size of 18.4 nm, as shown in Fig. S1.† The size distribution of particles ranges from 9 to 20.5 nm.

### 3.5 UV-vis/DRS

**Fig. 5:**
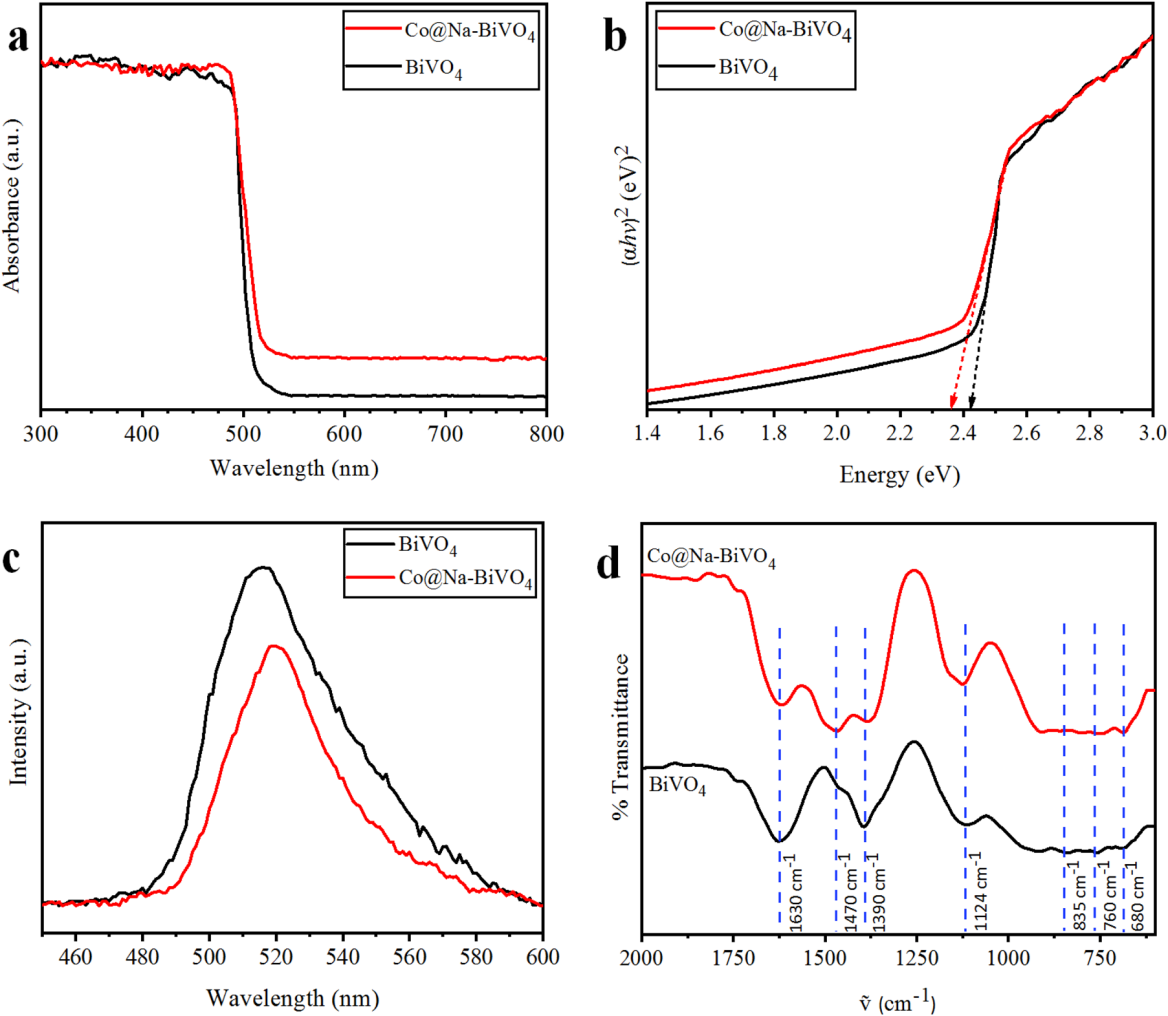
(a) UV-vis/DRS, (b) energy diagram, (c) PL spectra (both catalysts at the same concentration in ethanol) and (d) FT-IR spectra of the as-synthesized BiVO_4_ and Co@Na–BiVO_4_ (powders compressed into KBr pellets).

To evaluate the optical properties and band gap, UV-vis/DRS results of the as-synthesized photocatalysts were obtained (Fig. 5(a)). Plain BiVO_4_ shows maximum absorption at 512 nm, corresponding to its optical band gap *E*_g_ = 2.42 eV. For the Co@Na–BiVO_4_ microstructure, the absorption edge is slightly red-shifted to 525 nm (*E*_g_ = 2.36 eV), showing an excellent visible light response. The absorption of the Co@Na–BiVO_4_ microstructure further extends to 800 nm, which is due to Co d–d transitions^31^ and synergism of metal cocatalysts over BiVO_4_.^32^

### 3.6 PL

It is worth mentioning that our photoluminescence results confer the essential information about excitation, trapping and transfer of the charge carriers (e^−^ & h^+^). Higher recombination of e^−^ and h^+^ will result in higher emission intensities of PL signals.^33^ In this work, the high PL intensities (Fig. 5(c)) indicate a higher rate of charge recombination. These results indicate the higher charge separation in Co@Na–BiVO_4_ as compared to that in bulk BiVO_4_. As clearly seen in Fig. 5(c), Co@Na–BiVO_4_ intensity is weaker than that of BiVO_4_ indicating that recombination of charges is suppressed due to the presence of Co and Na metal oxides.

### 3.7 FT-IR

The FT-IR results of as-synthesized BiVO_4_ and Co@Na–BiVO_4_ microstructures exhibited the intense and broad absorption bands (Fig. 5(d)), due to V–O vibration at 760 cm^−1^ and 835 cm^−1^, respectively, whereas Bi–O bending vibration was observed at 680 cm^−1^. Additional bands are observed at 1390 cm^−1^ and 1470 cm^−1^, which are characteristic of stretching vibrations of the absorbed atmospheric and adventitious CO_2_ of the instrument. The band at 1630 cm^−1^ corresponds to the bending vibration of water due to the moisture content in KBr used in sample preparation for FT-IR analysis. In the FT-IR spectrum of Co@Na–BiVO_4_, Co and Na metal content slightly narrowed the typical absorption band.^34^

### 3.8 Photocatalytic degradation studies of MB, CR and RhB

**Fig. 6:**
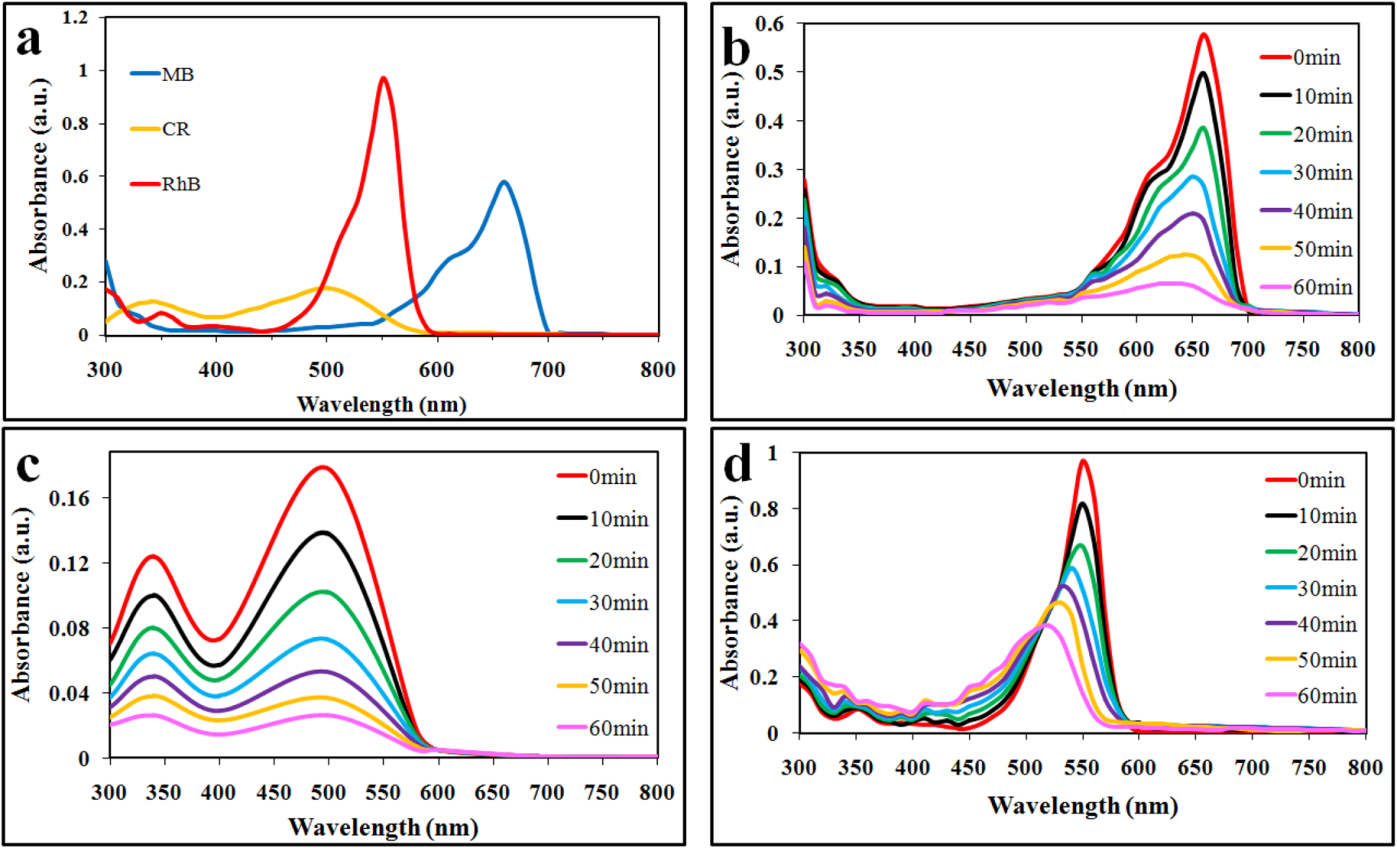
UV-vis spectrophotometry results: (a) lambda max, (b) MB, (c) CR and (d) RhB obtained over Co@Na–BiVO_4_ photocatalysts.

**Fig. 7:**
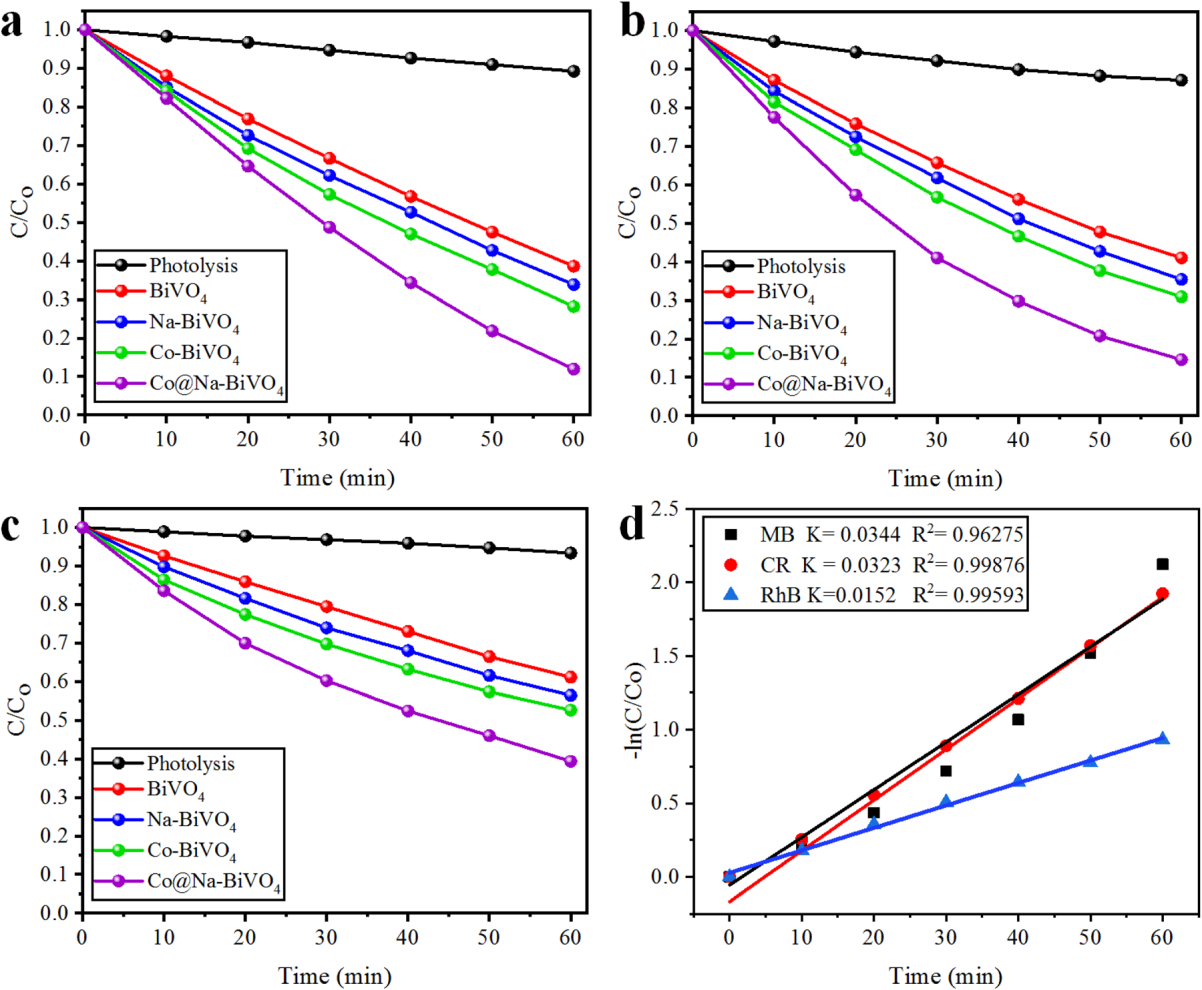
Comparative degradation activities of dyes with photolysis (degradation *via* photons), BiVO_4_, Na–BiVO_4_, Co–BiVO_4_ and Co@Na–BiVO_4_ (a) MB, (b) CR and (c) RhB, whereas (d) represents the kinetic study for MB, CR and RhB obtained over Co@Na–BiVO_4_ photocatalysts.

Photocatalytic dye degradation efficiencies are compared and quantified on the basis of the measured absorbance. It is important to select a suitable wavelength at which the dye solution shows the maximum absorbance. Using the Beer–Lambert law, the amount of dye was deliberated quantitatively with the absorption of light at *λ*_max_ (specific to each dye).^35^ The absorbance of dyes was observed to decrease during the photocatalytic reaction, indicating photocatalytic degradation. The efficiencies of photocatalytic degradation of each dye are determined by using the following formula:
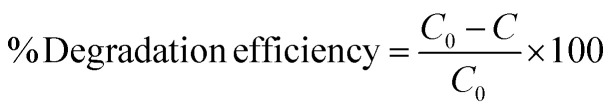
where *C*_0_ and *C* represent the concentrations before and after irradiation, respectively. In this work, MB, CR, and RhB dyes were selected for photocatalytic performances of the synthesized catalysts. For the photoreactions, 5 ppm concentration of each dye was optimized. For MB, CR, and RhB dyes, the maximum absorbance was measured at wavelengths of 664, 498 and 552 nm, respectively (Fig. 6(a)). The absorbance peak of MB at 664 nm is due to its azo groups as well as π–π* electronic excitations.^36^ The UV-vis absorption of CR exhibits two dominant absorption peaks observed at 347 and 498 nm, and a peak observed at 347 nm, attributed to the predominant absorption of a benzene group.^37,38^ The peak at 498 nm is due to the naphthalene group.^39^ Similarly, the prime peak for RhB is attributed to the combined effect of four *N*-ethyl groups in the xanthene ring structure.^40^ Based on our UV-vis results (Fig. 6(b and c)), it is confirmed that MB, CR, and RhB were degraded with efficiencies of 88%, 85% and 60%, respectively. Overall, higher photocatalytic degradation activities over Co@Na–BiVO_4_ were recorded for MB and CR dyes. The degradation of RhB was found to be less than 60%, due to the stable configuration of the xanthene ring. The blue shift indicates the de-ethylation and organic intermediates produced during the photoreaction.^41^ Photocatalytic activities of as-synthesized Co@Na–BiVO_4_ were compared with those of Co–BiVO_4_, Na–BiVO_4_, bulk BiVO_4_ and photolysis as well (*i.e.* photoreaction of dyes without any catalyst) as shown in Fig. 7(a–c). The specialty of Co@Na–BiVO_4_ is that it gives the highest degradation activity (88%) than previously reported works (see Table S2†). Similarly, 85% efficiency was noted for the CR dye that is almost slightly less than that in the case of MB. BiVO_4_ gives 60% activity for CR and direct photolysis exhibits 13% only. Co@Na–BiVO_4_ showed 60% degradation efficiency for RhB, whereas BiVO_4_ (*i.e.* without Co & Na) exhibits 39% only. It has been observed that Na–BiVO_4_ is active and gives 64.6% degradation individually for the CR dye, whereas the Co–BiVO_4_ catalyst exhibits 69.1% activity for CR. The catalysts having both Co and Na contents exhibit 85% dye degradation activity that is almost two times higher than that of BiVO_4_ or that of individual Co/Na over BiVO_4_ surfaces. These results demonstrate that the existence of Co in the form of Co_3_O_4_ over BiVO_4_ is more favorable than the existence of alkali metal oxides (*i.e.* Na_2_O), because due to Na and Co, synergism was established for charge transportation to active sites. Due to synergism, electrons and holes can build equilibrium for migration and transfer from the support to active sites. On the basis of photocatalytic activities, it can be concluded that the presence of Co and Na oxides offers an effective synergism during the photoreaction. Table S1† demonstrates the comparison of the efficiencies of as-synthesized Co@Na–BiVO_4_ (the most active) with those of the reported photocatalysts. Fig. 7(d) demonstrates the photocatalytic apparent rate constant *k* for the degradation of MB, CR and RhB dyes using the Co@Na–BiVO_4_ catalyst.

### 3.9 Recyclability of the photocatalyst

**Fig. 8:**
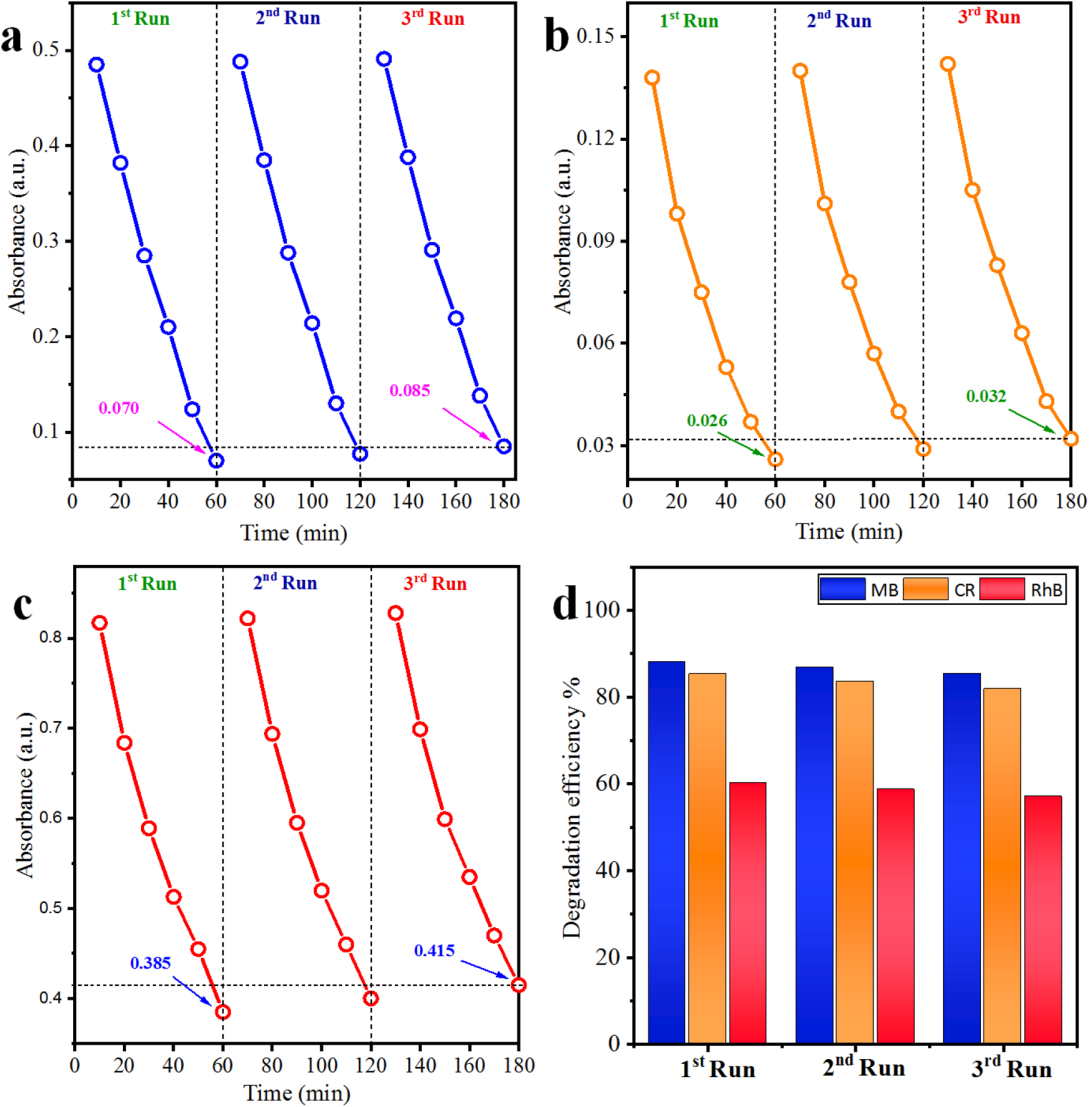
Recyclability of the Co@Na–BiVO_4_ catalyst for (a) MB, (b) CR and (c) RhB, whereas (d) represents the overall% degradation efficiency.

The recyclability tests of Co@Na–BiVO_4_ microstructures were further investigated (Table S3† and Fig. 8(a–d)). The results of most active photocatalysts demonstrate the highest stability (each run: up to 60 min for each cycle), and only a small loss of 2.6%, 3.4%, 3.1% was observed for MB, CR and RhB, respectively. The slight decrease in efficiency is actually due to the deposition of the photocatalyst particles on the reactor wall, which causes the loss of active sites. Thus, results obtained during recyclability tests assure the stability and continuous use of Co@Na–BiVO_4_ for dye degradation and other photocatalytic applications as well.

### 3.10 Effect of pH

**Fig. 9:**
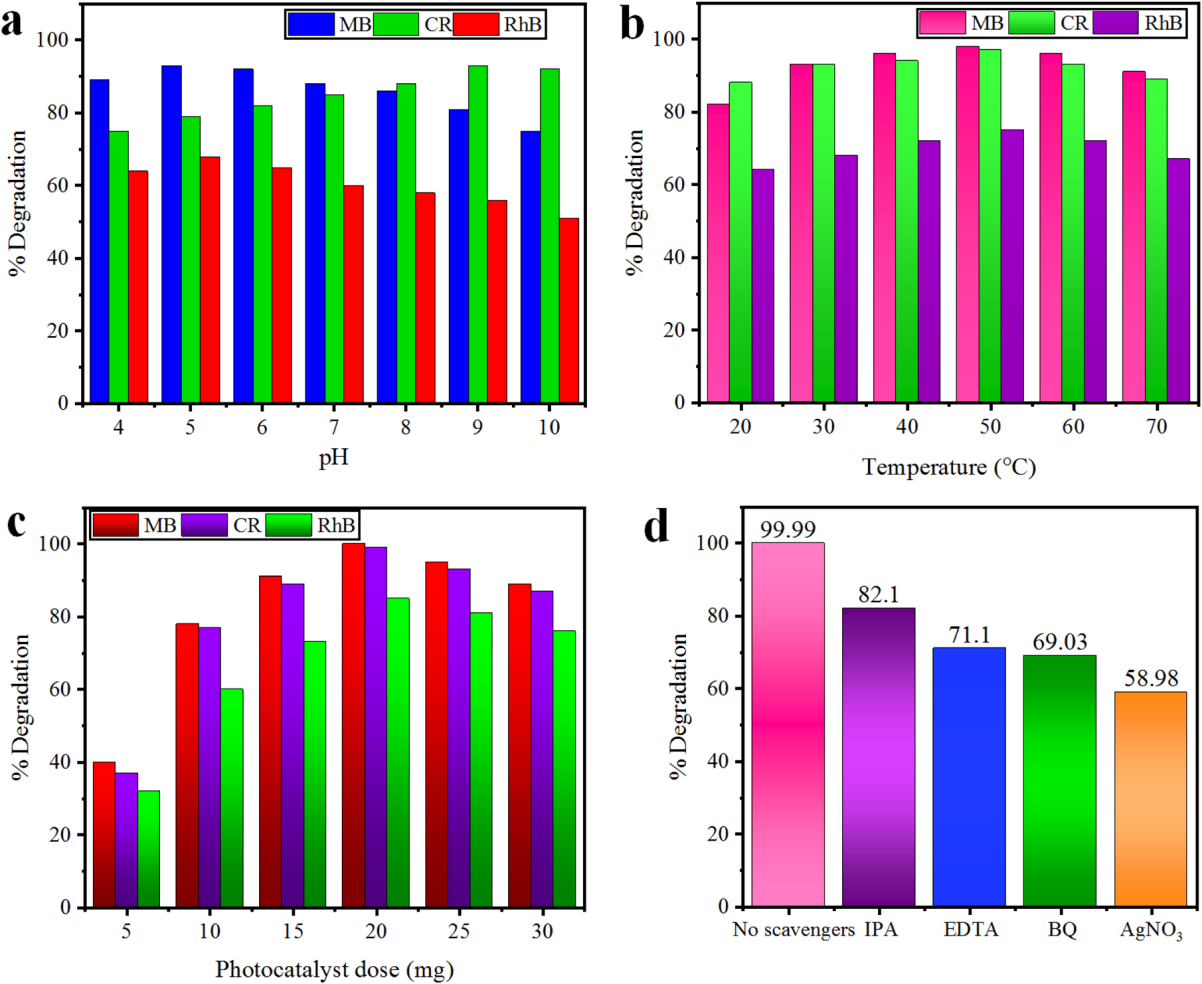
Effect of (a) pH, (b) temperature and (c) photocatalyst dose on MB, CR, and RhB dyes using the Co@Na–BiVO_4_ catalyst, whereas (d) represents the role of major active species.

The pH of the dye solution greatly affects the degradation of cationic (MB and RhB) as well as anionic (CR) dyes. The effect of pH was investigated in the range of 4–10 for our selected dyes using 10 mg of the Co@Na–BiVO_4_ catalyst for each photoreaction. The results for percentage dye removal are illustrated in Fig. 9(a). The results show that degradation of cationic (MB and RhB) dyes is increased under slightly acidic conditions due to their strong affinities with the surface of the catalyst; however, at below pH 5, photocatalytic degradation is inhibited by reducing the number of available electrons. Moreover, anionic (CR) dye degradation is increased at a slightly basic pH, while under highly basic conditions (>9), the formation of hydroxyl radicals hinders the degradation process by reducing the number of available holes.^42^

### 3.11 Effect of temperature

The optimized pH for each dye solution was used to evaluate the effect of temperature during the photodegradation reaction. The 20–70 °C temperature range was set using 10 mg of the Co@Na–BiVO_4_ catalyst for each photoreaction. The results indicate that 50 °C (±2) is the optimum temperature that facilitates the effective collisions of photocatalysts and dyes molecules. When the temperature is further increased, the adsorption of dye molecules on the catalyst becomes less which reduced the photodegradation efficiency.^43^ In contrast, the results (Fig. 9(b)) illustrate that at low temperature, slower desorption and less collisions between dye molecules inhibits the photoreaction.

### 3.12 Effect of the photocatalyst dose

The photocatalyst doses have significant effect on the degradation of dyes; the effect of the dose was monitored using 5.0–30 mg Co@Na–BiVO_4_, at an optimized temperature and pH for each dye. The results illustrated in Fig. 9(c) prove that a high photocatalyst dose leads to the generation of more active sites and active species (˙O_2_^−^ and ˙OH), which results in a higher degradation rate.^44^ However, addition of more than 20 mg of photocatalysts has negative effects on the photocatalytic degradation process. A high dose can initiate the formation of photocatalyst aggregates, which reduces the available surface area, blocks sunlight, and reduces efficiency. In addition to this, a higher photocatalyst dose can cause increased costs, and environmental concerns. Consequently, 20 mg was chosen as the optimal catalyst amount using a 150 mL Pyrex reactor.

### 3.13 Role of sacrificial agents

To investigate the role of sacrificial agents, radical quenching experiments were carried out using the Co@Na–BiVO_4_ photocatalyst, and MB dye was chosen. Radical scavenging agents used during this study are *p*-benzoquinone (BQ, 0.001 mol L^−1^ for ˙O_2_^−^ radicals), isopropyl alcohol (IPA, 0.01 mol L^−1^ for ˙OH), AgNO_3_ (0.001 mol L^−1^ for e^−^) and ethylene diamine tetra acetate (EDTA, 0.01 mol L^−1^ for h^+^).^45^ The degradation efficiency was considerably suppressed by using BQ, EDTA, and IPA, as illustrated in Fig. 9(d). These results demonstrate that e^−^, ˙O_2_^−^ and h^+^ are the major active species. However, the addition of AgNO_3_ decreases the activities only a little, which discloses that e^−^ plays a very minor role in dye degradation.

### 3.14 Mechanism of the photoreaction

**Scheme 1:**
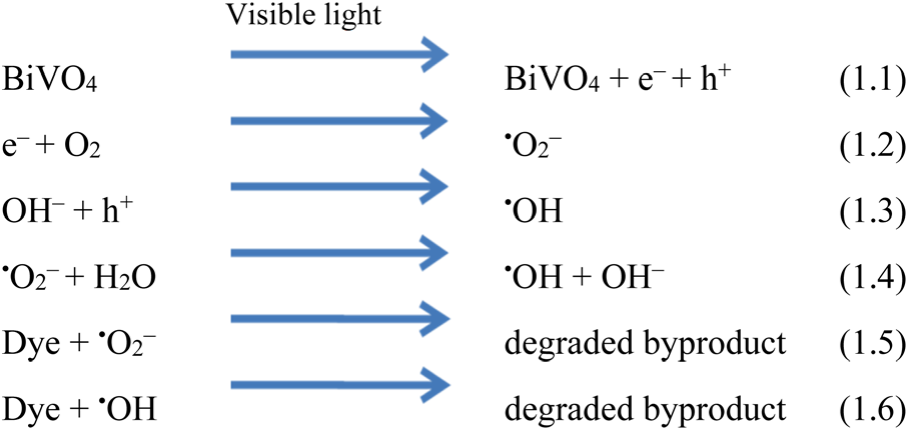
Photocatalytic reactions involved in dye degradation.

**Fig. 10:**
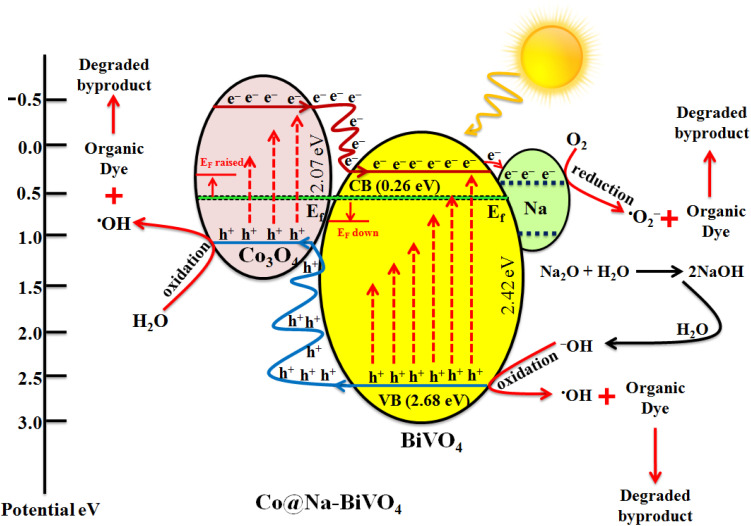
Synergism and dye degradation on the Co@Na–BiVO_4_ photocatalyst.

Understanding the mechanism is of vital importance in order to understand the overall efficiencies of photocatalyts.^46^ For the efficiency of any photocatalyst, it is essential to have a band gap less than 3 eV in order to extend light absorption and harvest maximum solar energy.^47^ It has been observed that the synergism between Co and Na enhances the redox sites for dye degradation due to their inherent characteristics during the photoreaction. The mechanism, band energy levels and charge transport (h^+^/e^−^) due to synergism over Co@Na–BiVO_4_ photocatalysts are illustrated in Fig. 7 and Scheme 1. The dye degradation results show that bare BiVO_4_ catalysts (*i.e.* in the absence of Co/Na) are not capable of transporting the photo induced charges to the active centres. Co_3_O_4_ is a p-type semiconductor, and when it is assembled with n-type BiVO_4_, the Fermi energy of both semiconductor systems aligned and shifted to new energy levels. Fermi level (*E*_F_) of Co_3_O_4_ lies near to the valence bands. During photoreaction, it gets a new position (raised) that makes the charge transportation more convenient.^48^ The *E*_F_ of BiVO_4_ present near the conduction band shifted down till equilibrium. When the photoreaction starts, electron–hole pairs started to migrate to photocatalyst surfaces where they are actually involved in the photodegradation of dyes. Due to higher CB levels of Co_3_O_4_ compared to those of BiVO_4_, the photogenerated e^−^ of Co_3_O_4_ preferably transfer to the CB of BiVO_4_ support. Electrons are further trapped by Na dopants where these are utilized by dissolved O_2_ to generate superoxide anion radicals (˙O_2_^−^),^23^ and thus, synergism starts to be established between Co/Na and the BiVO_4_ support. Meanwhile, holes (h^+^) migrate from the valence band of BiVO_4_ to the valence band of Co_3_O_4_, where they react with water molecules to generate ˙OH radicals. These ˙OH radicals are further utilized for dye degradation reactions. Overall higher dye degradation results were obtained in the presence of both Co & Na contents, because Na in the form of Na_2_O, when reacted with water produces NaOH (alkali). This NaOH increases the concentration of hydroxyl groups (^−^OH). The hydroxyl groups are further consumed by the holes at the surfaces of BiVO_4_ supports.^49,50^ The holes behave as electrophiles that convert –OH groups into radicals (˙OH). It has been observed that due to the presence of Na contents, a higher concentration of ˙OH is inevitable that is impossible in the case of bare BiVO_4_ or Co–BiVO_4_. The higher number of ˙OH radicals lead to the increased degradation of dyes that is only possible due to the existence of Na contents. In the presence of synergy, electrons of cobalt oxides migrated to the BiVO_4_ system and are trapped by doped Na, whereas holes migrate from BiVO_4_ to the Co_3_O_4_ system. Due to the synergistic effect, photocatalytic performance of BiVO_4_ has been increased mainly by (a) higher charge transportation to the active centres, (b) higher absorption of visible light, (c) efficient charge separation and (d) availably of more active sites (˙OH) due to Na. Without co-catalysts (Co and Na), BiVO_4_ is not efficient for dye degradation. Overall, as-synthesized Co@Na–BiVO_4_ leads to more active sites, which enhances the photocatalytic activity (Fig. 10).

## 4. Conclusion

Blossom-like BiVO_4_ microstructures have been successfully synthesized by the co-precipitation method with an optimized timescale strategy. Co and Na were *in situ* incorporated into BiVO_4_*via* low temperature chemical reduction followed by calcination. Powdered XRD and EDX analyses confirmed the existence of incorporated Co & Na over the BiVO_4_ support. The Raman and FT-IR results revealed the presence of active centers where dye degradation reactions occur. The UV-vis/DRS results revealed an extension in absorption to the visible spectrum, that is, from 512 nm to 530 nm due to Co/Na contents. The PL results indicate the suppressed recombination of photo-induced charges that is due to the synergism and continuous transfer of charges to active sites. Co@Na–BiVO_4_ exhibits a higher photocatalytic activity as compared to BiVO_4_, Co–BiVO_4_ or Na–BiVO_4_. Overall, the higher activities are attributed to synergistic charge transfer. Thus, the introduction of Co & Na in this work offers a new approach for photocatalysis applications. Furthermore, the recyclability test assures the structural stability of the assynthesized catalysts. Although various morphologies of BiVO_4_ have been designed for dye degradation, the introduction of Co and Na contents over blossom-like BiVO_4_ microstructures delivers higher dye degradation activities.

## Conflicts of interest

The authors declare no competing financial interest.

## Supplementary Material

NA-005-D3NA00048F-s001
