# Supplementary material for: Synergism of Co/Na in BiVO4 microstructures for visible-light driven degradation of toxic dyes in water
Source: Nanoscale Adv. 2023 Mar 27;5(12):3247–59. doi: 10.1039/d3na00048f (PMC10262974; doi:10.1039/d3na00048f)
Supplement: NA-005-D3NA00048F-s001 [file NA-005-D3NA00048F-s001.pdf]

Electronic Supplementary Material (ESI) for Nanoscale Advances.
This journal is © The Royal Society of Chemistry 2023

Supporting information

Synergism of Co/Na in $BiVO_4$ microstructures for visible-light driven degradation of toxic dyes in water†

Muhammad Zeeshan Abid[a], Khezina Rafiq[a], Abdul Rauf[a], Syed Shoaib Ahmad Shah[b], Rongchao Jin[c]*, Ejaz Hussain[ac]*

[a] Inorganic Materials Laboratory 52S, Institute of Chemistry, The Islamia University of Bahawalpur-63100, Pakistan

[b] Department of Chemistry, School of Natural Sciences, National University of Sciences and Technology, Islamabad-24090, Pakistan.

[c] Department of Chemistry, Carnegie Mellon University, Pittsburgh, Pennsylvania-15213, United States

Section 1: Experimental

Synthesis of Na-$BiVO_4$: For the synthesis of Na-$BiVO_4$, an optimized amount of $BiVO_4$ (~175 mg) powder was transfer into a three necks round bottom flask followed by addition of 50 mL of high purity deionized water (PIAS-GW1-Z) to produce the slurry. For depositing the Na, optimized amount of Na^+ ions (NaOH solution) were introduced into $BiVO_4$ slurry stirrer for 5 h followed by 30 min sonication. The precipitate of Na-$BiVO_4$ was filtered using vacuumed filtration and finally the product was calcined at 350 °C for 3 h to converts Na^+ into Na_2O.

Synthesis of Co-$BiVO_4$: The synthesis of Co-$BiVO_4$ microstructures, an optimized amount of $BiVO_4$ (~255 mg) powder was transfer into a three necks round bottom flask followed by addition of 50 mL of high purity deionized water to produce uniform suspension. This suspension was then purge with high purity argon gas, to deposit the Co, $Co(NO_3)_2.6H_2O$ solution were introduced into $BiVO_4$ slurry. The Co metal ions were then in-situ reduced over $BiVO_4$ using freshly prepared cold alkaline borohydride solution (~10 °C). The final suspension was filtered, thoroughly washed and dried at 94 °C. The product was calcined at 350 °C for 3 h.

Cobalt reduction: It is reported that, reducing the Co with $NaBH_4$ generate proton (H^+) that promotes the hydrolysis of borohydride. The existence of NaOH that produce the hydroxyl ions ^-OH further favors the cobalt reduction. Moreover, the efficiency of cobalt reduction increases with decreasing temperature of borohydride solution and increasing pH, which neutralizes the released H^+ during cobalt reduction [1, 2].

Table S1: EDX Analysis Weight and Atomic percentage of elements in as-synthesized $Co@Na\text{-}BiVO_4$.

Elements	Weight %	Atomic %
Bi	55.81	11.85
V	16.31	13.95
O	23.62	64.30
Co	1.29	0.95
Na	1.16	2.26
C	1.81	6.69
Totals	100.00	

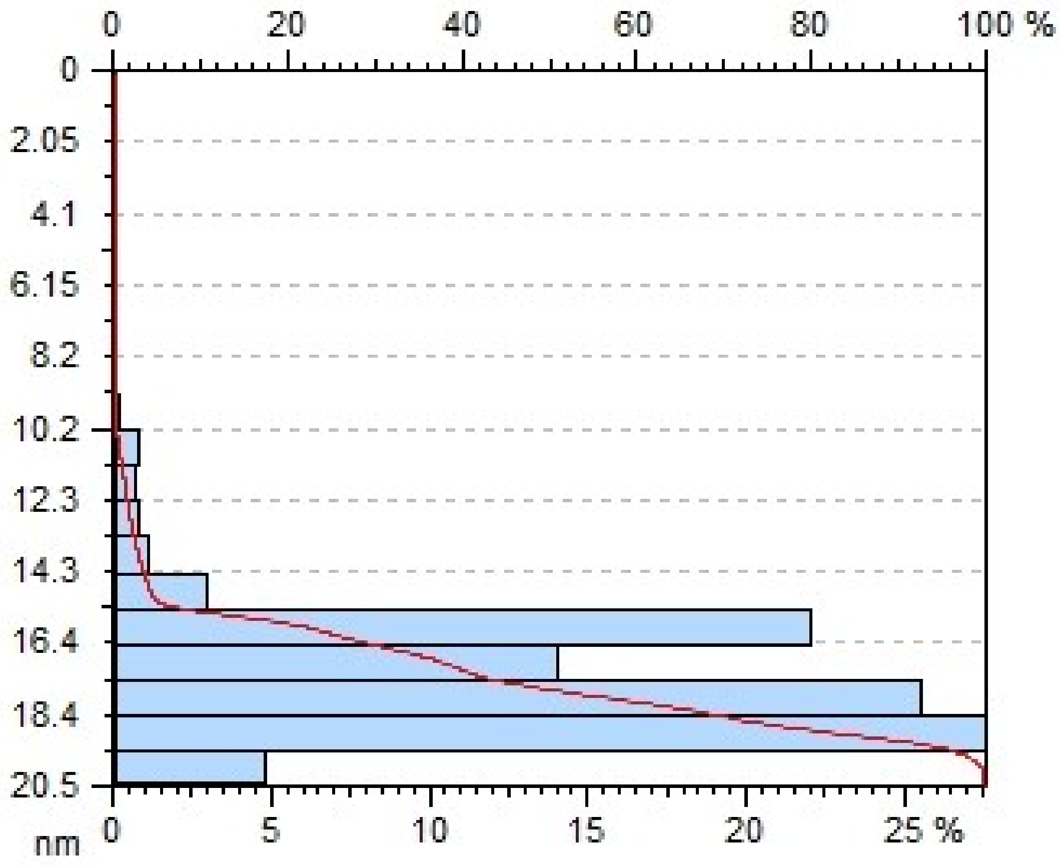

Figure S1: Particle size distribution of $Co@Na\text{-}BiVO_4$.

Table S2: Comparative analysis of MB, CR, and RhB dye degradation efficiency of previously reported metal doped-photocatalysts with currently synthesized Co@Na-$BiVO_4$ photocatalysts.

Materials	Dye	% efficiency	Time (min)	(%/min) efficiency	Source	Ref.
$BiVO_4$	MB	50 %	120 min	0.41	UV-Visible	[3]
Paint coated $BiVO_4$	MB	72 %	240 min	0.30	Visible	[4]
$BiVO_4/TiO_2$	MB	85 %	120 min	0.70	Visible	[5]
Polycrystalline $BiVO_4$	CR	55 %	60 min	0.91	Visible	[6]
m-$BiVO_4$	RhB	64.8 %	210 min	0.31	Visible	[7]
$BiVO_4$ (biscuits)	RhB	68 %	270 min	0.25	Visible	[8]
ZnO/$BiVO_4$	RhB	72.7 %	150 min	0.48	Visible	[9]
TiO_2-$BiVO_4$	RhB	79.3 %	300 min	0.26	Sunlight	[10]
Co@Na-$BiVO_4$	MB	88 %	60 min	1.46	Sun-light	Current work
Co@Na-$BiVO_4$	CR	85 %	60 min	1.41	Sun-light	Current work
Co@Na-$BiVO_4$	RhB	60 %	60 min	1.0	Sun-light	Current work

Table S3 Recyclability test of $Co@Na\text{-}BiVO_4$ microstructures for degradation of MB, CR and RhB dyes

Dye	Photocatalyst $Co@Na\text{-}BiVO_4$	Absorbance of selected dyes with time intervals						Degradation efficiency (%)
		Time (min)						
		10	20	30	40	50	60	
MB	1st Run	0.485	0.382	0.285	0.210	0.124	0.07	88 %
	2nd Run	0.488	0.385	0.288	0.214	0.130	0.077	86.8 %
	3rd Run	0.491	0.388	0.291	0.219	0.138	0.085	85.4 %
CR	1st Run	0.138	0.102	0.073	0.053	0.037	0.026	85.3 %
	2nd Run	0.140	0.105	0.078	0.057	0.040	0.029	83.6 %
	3rd Run	0.142	0.105	0.083	0.063	0.043	0.032	81.9 %
RhB	1st Run	0.817	0.684	0.589	0.513	0.455	0.385	60.2 %
	2nd Run	0.822	0.694	0.595	0.520	0.460	0.400	58.7 %
	3rd Run	0.828	0.699	0.599	0.535	0.47	0.415	57.1 %

Note: Absorbance was measured at the λ-max of each dye.

References:

1. Lu, J., D. Dreisinger, and W. Cooper, *Cobalt precipitation by reduction with sodium borohydride.* Hydrometallurgy, 1997. **45**(3): p. 305-322.
2. Glavee, G., et al., *Sodium borohydride reduction of cobalt ions in nonaqueous media. Formation of ultrafine particles (nanoscale) of cobalt metal.* Inorganic chemistry, 1993. **32**(4): p. 474-477.
3. Obregón, S., A. Caballero, and G. Colón, *Hydrothermal synthesis of BiVO4: Structural and morphological influence on the photocatalytic activity.* Applied Catalysis B: Environmental, 2012. **117-118**: p. 59-66.
4. Kumar, M. and R. Vaish, *Photocatalytic dye degradation using BiVO4–paint composite coatings.* Materials Advances, 2022. **3**(14): p. 5796-5806.
5. Wetchakun, N., et al., *Efficient photocatalytic degradation of methylene blue over BiVO4/TiO2 nanocomposites.* Ceramics International, 2015. **41**(4): p. 5999-6004.
6. Madhusudan, P., et al., *Hydrothermal synthesis of meso/macroporous BiVO4 hierarchical particles and their photocatalytic degradation properties under visible light irradiation.* Environmental Science and Pollution Research, 2013. **20**(9): p. 6638-6645.
7. Sajid, M.M., et al., *Hydrothermal fabrication of monoclinic bismuth vanadate (m-BiVO4) nanoparticles for photocatalytic degradation of toxic organic dyes.* Materials Science and Engineering: B, 2019. **242**: p. 83-89.
8. Lu, Y., et al., *Novel core–shell structured BiVO4 hollow spheres with an ultra-high surface area as visible-light-driven catalyst.* CrystEngComm, 2014. **16**(27): p. 6059-6065.
9. Singh, S., et al., *Formation of intermediate band and low recombination rate in ZnO-BiVO4 heterostructured photocatalyst: Investigation based on experimental and theoretical studies.* Korean Journal of Chemical Engineering, 2017. **34**(2): p. 500-510.
10. Wang, Y., et al., *Enhancement mechanism of fiddlehead-shaped TiO2-BiVO4 type II heterojunction in SPEC towards RhB degradation and detoxification.* Applied Surface Science, 2019. **463**: p. 234-243.
